# Repeated cycles of chronic intermittent ethanol exposure increases basal glutamate in the nucleus accumbens of mice without affecting glutamate transport

**DOI:** 10.3389/fphar.2015.00027

**Published:** 2015-02-23

**Authors:** William C. Griffin, Vorani S. Ramachandra, Lori A. Knackstedt, Howard C. Becker

**Affiliations:** ^1^Charleston Alcohol Research Center, Department of Psychiatry and Behavioral Sciences, Medical University of South Carolina, Charleston, SC, USA; ^2^Department of Psychology, University of Florida, Gainesville, FL, USA; ^3^Department of Neurosciences, Medical University of South Carolina, Charleston, SC, USA; ^4^Ralph H. Johnson VA Medical Center, Charleston, SC, USA

**Keywords:** alcohol, mouse, microdialysis, uptake, transport

## Abstract

Repeated cycles of chronic intermittent ethanol (CIE) exposure increase voluntary consumption of ethanol in mice. Previous work has shown that extracellular glutamate in the nucleus accumbens (NAc) is significantly elevated in ethanol-dependent mice and that pharmacologically manipulating glutamate concentrations in the NAc will alter ethanol drinking, indicating that glutamate homeostasis plays a crucial role in ethanol drinking in this model. The present studies were designed to measure extracellular glutamate at a time point in which mice would ordinarily be allowed voluntary access to ethanol in the CIE model and, additionally, to measure glutamate transport capacity in the NAc at the same time point. Extracellular glutamate was measured using quantitative microdialysis procedures. Glutamate transport capacity was measured under Na^+^-dependent and Na^+^-independent conditions to determine whether the function of excitatory amino acid transporters (also known as system X_AG_) or of system X_c_^–^ (glial cysteine–glutamate exchanger) was influenced by CIE exposure. The results of the quantitative microdialysis experiment confirm increased extracellular glutamate (approximately twofold) in the NAc of CIE exposed mice (i.e., ethanol-dependent) compared to non-dependent mice in the NAc, consistent with earlier work. However, the increase in extracellular glutamate was not due to altered transporter function in the NAc of ethanol-dependent mice, because neither Na^+^-dependent nor Na^+^-independent glutamate transport was significantly altered by CIE exposure. These findings point to the possibility that hyperexcitability of cortical–striatal pathways underlies the increases in extracellular glutamate found in the ethanol-dependent mice.

## INTRODUCTION

Prolonged excessive alcohol (ethanol) consumption can lead to dependence, a condition characterized by many neuroadaptive changes in brain reward and stress systems (Hansson et al., 2008; Koob and Le Moal, 2008; Spanagel, 2009). These neuroadaptive changes trigger withdrawal symptoms when drinking is terminated, increase vulnerability to relapse, and facilitate a shift from regulated drinking to less controlled and more excessive ethanol consumption (Becker, 2008; Vengeliene et al., 2009; Heilig et al., 2010). In particular, an adaptive up-regulation of glutamatergic activity following chronic ethanol treatment is well-documented in animal studies (Gass and Olive, 2008). For example, in rodents, microdialysis studies have revealed elevated extracellular levels of glutamate following chronic ethanol treatment in several brain regions including dorsal striatum, nucleus accumbens (NAc), hippocampus, and the ventral tegmental area (Dahchour et al., 2000; Baker et al., 2002; Dahchour and De Witte, 2003; Melendez et al., 2005; Ding et al., 2012, 2013; Griffin et al., 2014). Similarly, using magnetic resonance spectroscopy (MRS) techniques in rodents, increased glutamate activity has been reported in prefrontal cortex (Hermann et al., 2012) and basal ganglia (Zahr et al., 2009; Gu et al., 2014). Emerging evidence indicates similar findings in human alcoholics (Hermann et al., 2012; Bauer et al., 2013). Thus, across different model systems and procedures, chronic exposure to ethanol produces increased glutamatergic activity.

We have developed a mouse model of ethanol dependence and relapse drinking that involves chronic intermittent ethanol (CIE) exposure and produces significant escalation of voluntary ethanol consumption in dependent compared to non-dependent mice (Becker and Lopez, 2004; Lopez and Becker, 2005; Griffin et al., 2009b, 2014; Griffin, 2014). Recently, using *in vivo* microdialysis techniques, we reported that ethanol-dependent mice have increased extracellular glutamate concentrations in the NAc (Griffin et al., 2014). Importantly, this effect was shown to be sustained beyond acute withdrawal (at least 6–7 days following CIE exposure). Further, pharmacologically increasing or decreasing glutamatergic concentrations in the accumbens increased or decreased, respectively, ethanol drinking in the model (Griffin et al., 2014). These findings indicate an important role for accumbal glutamatergic transmission in regulating ethanol drinking, and increased glutamate activity in the NAc following chronic ethanol exposure may contribute to escalated drinking associated with dependence. Further, our findings are consistent with other reports demonstrating a relationship between glutamate activity and the regulation of ethanol consumption in mice (Kapasova and Szumlinski, 2008; Szumlinski et al., 2008). Together, these results provide evidence for a significant role for glutamate in the addiction process (Kalivas and O’Brien, 2008).

Although it is known that extracellular glutamate levels are tightly regulated by numerous neuronal and glial functions (Danbolt, 2001), the mechanism underlying elevated glutamatergic activity in ethanol dependence is unknown. Active transporter mechanisms in neurons and glia play a critical role in maintaining glutamate homeostasis in the synapse. Glutamate transporters (excitatory amino acid transporters, EAATs) operate to remove glutamate from the extracellular space (synapse) in a sodium (Na^+^)-dependent manner (Danbolt, 2001). The glia-based system X_c_^–^ is Na^+^-independent and exchanges extracellular cysteine for intracellular glutamate, which contributes significantly to the extrasynaptic pool of glutamate (Baker et al., 2002). Interestingly, while previous work using rats indicates that non-contingent ethanol exposure increases glutamate concentrations in the accumbens, this was not associated with significant alterations in Na^+^-dependent glutamate transporter expression (Melendez et al., 2005). However, recent studies using voluntarily drinking P rats have found increases in glutamate in the NAc to be associated with decreases in Na^+^-dependent transporters, specifically EAAT1, but not EAAT2 or system X_c_^–^expression (Ding et al., 2013; Alhaddad et al., 2014a). The present study was conducted to confirm our earlier findings of increased extracellular glutamate levels in the following CIE exposure using quantitative microdialysis procedures, as well as to investigate whether CIE exposure produces alterations in glutamate transporter function that contributes to the observed increase in basal glutamate in ethanol-dependent mice.

## MATERIALS AND METHODS

### SUBJECTS

Male C57BL/6J mice (10–14 weeks) were obtained from Jackson Laboratories (Bar Harbor, ME, USA) and maintained in a temperature and humidity controlled AAALAC accredited facility under a 12 h light cycle (lights on 0200 h). Mice were initially group housed during a 2–4 week period of acclimation to the vivarium, and then individually housed for the remainder of the experiments. Food and water were available *ad libitum* at all times. All experimental procedures were approved by the Institutional Animal Care and Use Committee at the Medical University of South Carolina and were consistent with the guidelines of the NIH Guide for the Care and Use of Laboratory Animals. Separate cohorts of mice were utilized for the microdialysis and glutamate transport experiments.

### CHRONIC INTERMITTENT ETHANOL EXPOSURE

Chronic intermittent ethanol exposure was administered via inhalation using a well-established ethanol dependence model in mice (Becker and Lopez, 2004; Lopez and Becker, 2005; Griffin, 2014; Griffin et al., 2014). Briefly, one group of mice (ethanol-dependent; EtOH group) received chronic intermittent exposure to ethanol vapor in inhalation chambers (16 h/day for 4 days) while the remaining mice (non-dependent; CTL group) were similarly handled, but maintained in control (air) inhalation chambers. This pattern of CIE (or air) vapor exposure was repeated over 3 weekly cycles for the quantitative microdialysis study and 4 weekly cycles for the studies involving glutamate transport assays. In both studies, the weekly inhalation exposure cycles were alternated with intervening weeks when animals were left undisturbed in the colony room. Ethanol (95%) was volatilized, mixed with fresh air, and delivered to Plexiglas inhalation chambers at a rate set to yield blood ethanol levels in the range of 175–225 mg/dl. These values were verified by measuring chamber ethanol concentrations (daily) and blood ethanol concentrations (weekly) as previously described (Griffin et al., 2009a). Prior to being placed in the ethanol vapor chambers, EtOH mice were administered a loading dose of ethanol (1.6 g/kg; 8% w/v) and the alcohol dehydrogenase inhibitor pyrazole (1 mmol/kg) by intraperitoneal injection. CTL mice received injections of saline and pyrazole before being placed in control chambers. During the inhalation treatment, the housing conditions were identical to those in the colony room.

### MICRODIALYSIS GUIDE IMPLANT SURGERY

Surgical procedures were performed as previously described (Griffin et al., 2007, 2009b; Griffin, 2014). Briefly, mice were anesthetized with isoflurane gas (4% induction, 1.5% maintenance) and placed in a Kopf stereotaxic instrument with digital display (Model 942). Guide cannulae (10 mm long) were implanted just above the left (NAc) (coordinates relative to Bregma: AP +1.7, ML +0.8 and DV –3.5) and secured to the skull using a light-cured resin system. Guide obdurators remained in place until microdialysis procedures were initiated (see below). After surgery, mice were allowed at least 1 full week of recovery before experimental procedures commenced.

### QUANTITATIVE (NO NET FLUX) MICRODIALYSIS PROCEDURES

Microdialysis was conducted in EtOH and CTL groups at 72 h after the final CIE (or air) exposure cycle. On the day before microdialysis, mice were lightly restrained and microdialysis probes (CMA/7; CMA Microdialysis, Sweden) were inserted at least 16 h prior to sample collection (probes extended 1 mm beyond the guide cannulae). The aCSF consisted of: 140 mM NaCl; 7.4 mM Glucose; 3 mM KCl; 0.5 mM MgCl2; 1.2 mM CaCl2; 1.2 mM Na_2_HPO_4_; 0.3 mM NaH2PO_4_; pH = 7.4 and was filtered (0.22 μm) before use. aCSF was perfused through the probe at an overnight flow-rate of 0.2 μL/min, as previously described (Griffin et al., 2014). The flow rate was increased to 1.0 μL/min on the following day and sample collection commenced 2 h later. Dialysates were collected every 15 min and an aliquot (10 μL) of each sample was immediately frozen on dry ice and stored at –80°C until analysis. During the collection procedure, increasing concentrations of glutamate were added to the aCSF and perfused through the probes (0, 0.2, 1, 2.5, and 5 μM). Four samples were collected at each concentration and the last three samples from each series were averaged and analyzed for glutamate content. Glutamate concentrations were determined using high pressure liquid chromatography (HPLC) with fluorescence detection, as previously described (Griffin et al., 2014).

### HISTOLOGY

At the end of the quantitative dialysis experiment, mice were overdosed with urethane (1.5 g/kg i.p.), transcardially perfused, and brains were then extracted and stored in 10% formalin for a period of 3–5 days as previously described (Griffin et al., 2009b). The brains were sectioned at 50 μm and stained with cresyl violet to evaluate probe placement using a mouse brain atlas as a guide (Paxinos and Franklin, 2001).

### GLUTAMATE TRANSPORT ASSAY

At 72 h following the final CIE (or air) exposure cycle, mice were sacrificed via rapid decapitation, the brains were rapidly extracted, and 1 mm bilateral tissue punches of the NAc core were dissected on ice. To ensure an adequate amount of tissue was available for analysis, accumbal tissue samples were pooled from two mice in each condition. Using a McIllwan tissue chopper (St. Louis, MO, USA), the tissue was cut into 250 × 250 μm slices which then underwent three 10 min washes at 37Â°C using an oxygenated Krebs–Ringer’s solution (140 mM NaCl, 1.3 mM CaCl2, 1.2 mM KH_2_PO_4_, 5 mM HEPES, 10 mM glucose and 1 mM MgCl_2_; final pH 7.4). To measure glutamate uptake, L-[^3^H] glutamate (250 nM, 51 Ci/mM; Perkin-Elmer, Boston, MA, USA) was added to aliquots of tissue-slice samples in the presence of 0.1, 1, 10 100 and 1000 μM unlabeled L-glutamate in a final volume of 250 μL of oxygenated buffer. Na^+^-independent uptake was measured by conducting the final wash and slice incubation in buffer where NaCl was replaced with 140 mM choline chloride. All incubations were conducted in triplicate. After incubation at 37°C for 15 min, the reaction was terminated by washing the slices in ice-cold, Na^+^-free buffer. Slices were then solubilized using 1% sodium dodecyl sulfate at room temperature for 12 h and radioactive counts subsequently determined using a liquid scintillation counter (Packard 1900 TR). Protein content in the slices was determined using a bicinchoninic acid assay (Pierce Biotechnology, Inc., Rockford, IL, USA). Counts per minute were converted to uptake/mg protein/15 min.

To confirm Na^+^-dependent and -independent transport in this assay, a separate cohort of ethanol naïve animals (*n* = 3) were sacrificed and measurements conducted in the presence of the non-selective EAAT inhibitor TBOA (0, 10, or 50 μM) or the System X_c_^–^ inhibitor CPG (0, 5, or 25 μM), respectively. These experiments were conducted at the 10 μM glutamate concentration prepared in both Na^+^-containing and Na^+^-free buffers.

### CHEMICALS

DL-threo-β-benzyloxyaspartate (TBOA) and (S)-4-Carboxy-phenylglycine (CPG) were obtained from Tocris Bioscience, Inc., (Bristol, UK). The drugs were frozen in 2.5 mM and 50 mM stocks, respectively, in 1 × phosphate buffered saline (PBS) and diluted as needed. All other chemicals were purchased from Sigma-Aldrich, Inc (St Louis, MO, USA).

### DATA ANALYSES

The data from the quantitative microdialysis experiment were analyzed by first subtracting the known amount of glutamate added to the perfusate from the amount measured in the dialysate by HPLC analysis ([Glu]_in_ – [Glu]_out_) which is taken as the net flux of glutamate across the dialysis membrane. For individual mice, slopes of the linear function provide a measure of glutamate clearance and the X intercepts are taken to reflect basal glutamate concentration (Parsons and Justice, 1994). Therefore, these values were calculated for individual mice using the linear function (*y* = *mx* + *b*) and Student’s *t*-test was used to compare these measures between EtOH and CTL groups.

Glutamate transport analyses were conducted separately for the Na^+^-dependent and Na^+^-independent experiments. Non-linear curves, as shown in Figure 2, were generated using GraphPad, Prism 4 software (La Jolla, CA, USA). However, the data were analyzed using a mixed model procedure (SPSS^®^ version 18), with group and concentration as between-subject factors. Finally, for the TBOA and CPG uptake experiments, data were analyzed using analysis of variance with *post hoc* analysis using Bonferroni’s corrected *t*-test. For all analyses, alpha was set to 0.05.

## RESULTS

### QUANTITATIVE MICRODIALYSIS

**FIGURE 1:**
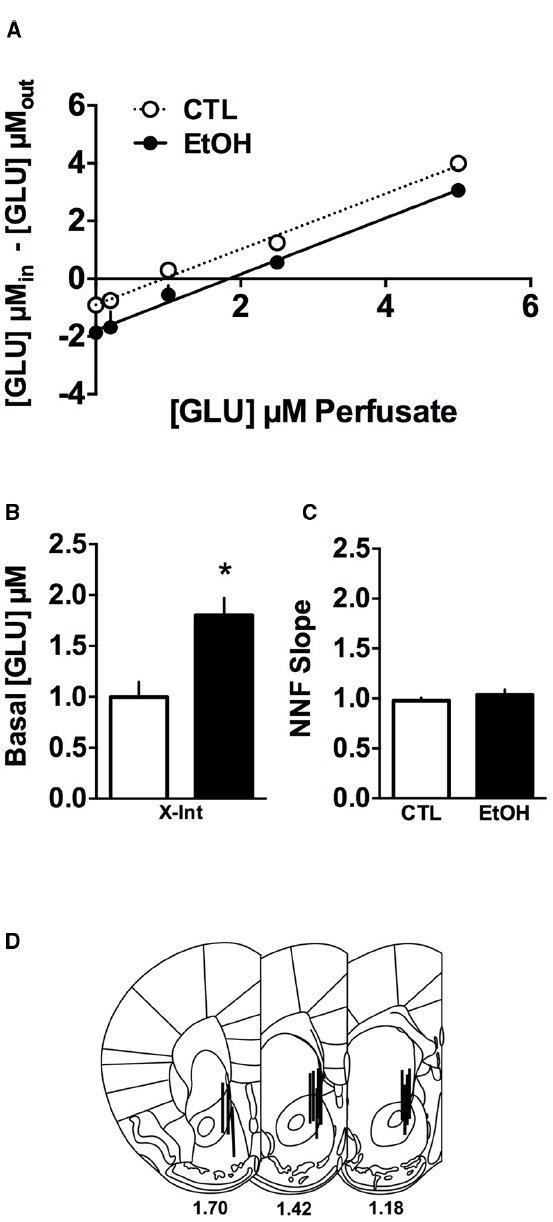
**Glutamate quantitative microdialysis in the nucleus accumbens (NAc) of ethanol-dependent (EtOH) and non-dependent (CTL) mice (*n* = 5–7/group). (A)** The group means and linear regressions on the dialysate glutamate levels are plotted for the no net flux function and indicate that the functions are parallel and there is a right shift for the ethanol-dependent mice, indicating higher basal levels of glutamate in the NAc of these mice. **(B)** After calculating the *x*-intercept for each mouse to determine basal glutamate concentrations use and comparison of the group means indicate that the basal glutamate concentrations were twofold higher (*p < 0.05). **(C)** The slopes of the no net function were calculated and the statistical comparison indicated no difference, consistent with the observation that the functions are parallel. **(D)** Probe placements for the mice included in the analyses. Data are means ± SEM.

A summary of results from the quantitative dialysis experiment (i.e., no net flux dialysis) for ethanol-dependent and non-dependent mice is shown in Figure 1A. As can be seen, the calculated X-intercept was greater for dependent (EtOH group) compared to non-dependent (CTL group) mice, indicative of higher basal glutamate concentrations in CIE-exposed mice. In contrast, the slopes of the linear functions for the two groups were parallel, suggesting that rate of glutamate uptake was similar for both groups. Individual x-intercepts, representing basal extracellular glutamate concentrations, and slopes of the linear function were calculated for mice in both groups and these data are summarized in Figures 1B,C, respectively. Ethanol-dependent mice exhibited significantly greater basal extracellular glutamate concentrations (*t*(10) = 3.362, *p* < 0.01) but the slopes of the non-net-flux function were not different (*t*(10) = 0.059, *p* > 0.05). Finally, Figure 1D shows the placements of the microdialysis probes.

### GLUTAMATE TRANSPORT

**FIGURE 2:**
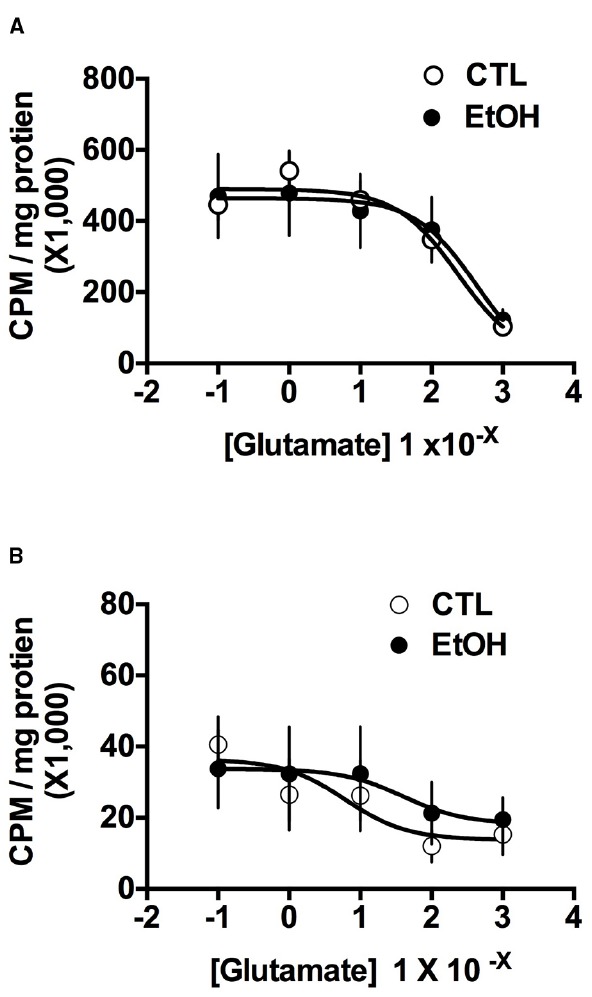
**Glutamate transport in NAc tissue from ethanol-dependent (EtOH) and non-dependent (CTL) mice (*n* = 6–7/group). (A)** Na^+^-dependent glutamate transport, which reflects the function of excitatory amino acid transporters in the cell membrane, was not influenced by ethanol dependence. **(B)** Na^+^-independent glutamate transport, which reflects the function of System X_c_^–^ in glial membranes, also was not affected by ethanol dependence. Data are means ± SEM.

Because regulation of extracellular glutamate concentrations is regulated by multiple processes (Winder and Conn, 1996; Danbolt, 2001), tritiated-glutamate uptake assays were used to further examine the influence of ethanol dependence on transporter function. Results of the glutamate transport assays are summarized in Figure 2. As can be seen, ethanol-dependent and non-dependent mice did not differ in either Na^+^-dependent (Figure 2A) or Na^+^-independent (Figure 2B) glutamate transport. These observations were supported by a mixed model analysis of the data. As expected, for the Na^+^-dependent transport assay, ANOVA indicated a significant main effect of glutamate Concentration [*F*(4,165) = 25.15, *p* < 0.0001], but there was no main effect of group [*F*(1,165) < 1] or an interaction of the group and concentration factors [*F*(1,165) < 1]. Similarly, for the Na^+^-independent transport experiment, ANOVA revealed a significant main effect of glutamate concentration [*F*(4,164) = 7.274, *p* < 0.0001], but no effect of group [*F*(1,164) = 1.160, *p* = 0.283] or the group × concentration interaction [*F*(4,164) = 2.079, *p* = 0.086]. These analyses indicate that increasing concentrations of glutamate significantly influenced glutamate transport (i.e., reaching saturation of the transport proteins) under both Na^+^-dependent and -independent conditions, but a history of ethanol dependence did not affect glutamate transport.

To confirm the specificity of the glutamate transport assay in mouse tissue, the ability of TBOA to decrease Na^+^-dependent transport and CPG to decrease Na^+^-independent transport were tested (Table 1). In both cases, data were expressed as cpm/mg protein/15 min and normalized to the zero concentration condition. As expected, TBOA reduced glutamate transport by ∼50% at the low concentration and ∼65% at the higher concentration, relative to the control condition. This was confirmed by one-way ANOVA (*F*(2,21) = 12.752, *p* < 0.001). *Post hoc* analysis indicated significant differences between transport at 0 μM TBOA and 10 μM TBOA and 50 μM TBOA (both *p* < 0.05). Similarly, in the Na^+^-independent condition, CPG significantly inhibited glutamate transport to ∼35% of control levels at both concentrations tested. One-way ANOVA on these data indicated a significant effect of dose (*F*(2,22) = 16.574, *p* < 0.001) and *post hoc* tests revealed differences between 0 μM CPG and the 5 μM CPG and 25 μM CPG conditions (both *p* < 0.05). These data provide confirmation that the glutamate transport assay is sensitive to changes in both Na^+^-dependent and Na^+^-independent glutamate transport functions.

**Table 1:** **Effects of TBOA and CPG on glutamate uptake**.

**Condition/inhibitor**	**Inhibitor (μM)**	**% of control**
Na^+^-dependent/TBOA	10	^*^55 ± 10
	50	^*^37.8 ± 11.5
Na^+^-independent/CPG	51	^*^32.6 ± 4
	25	^*^30.3 ± 7.3

n = 3; data are means ± SEM. *p < 0.05 versus control condition, normalized to 100%.

## DISCUSSION

Results from the *in vivo* quantitative dialysis experiment indicated that extracellular concentrations of glutamate in the NAc were increased approximately twofold in ethanol-dependent compared to non-dependent mice. This finding is consistent with our earlier work (Griffin et al., 2014) that reported a twofold increase in baseline glutamate concentrations in CIE-exposed mice using conventional dialysis procedures. The data presented here are also consistent with several other studies showing ethanol exposure increased basal glutamate levels in the NAc (Melendez et al., 2005; Kapasova and Szumlinski, 2008; Ding et al., 2013), as well as other brain regions including hippocampus (Moghaddam and Bolinao, 1994; Dahchour and De Witte, 1999; Chefer et al., 2011) and the amygdala (Roberto et al., 2004). While there are some studies using have not reported increases in glutamate concentrations following ethanol exposure (Szumlinski et al., 2005; Goulding et al., 2011), in general it appears that ethanol exposure consistently increases glutamatergic activity in several brain regions including the NAc.

Because we did not find differences between ethanol-dependent and non-dependent mice in the slopes of the linear function calculated in the microdialysis experiment, these results suggested that the difference in basal glutamate levels was not due to alterations in glutamate transport mechanisms as the slope has been suggested to be a measure of the extraction fraction (*E*_d_), which is potentially an estimate of neurotransmitter clearance (Bungay et al., 2003). But the slope has only been empirically demonstrated to be an indication of clearance rate for the neurotransmitters dopamine (Smith and Justice, 1994) and acetylcholine (Vinson and Justice, 1997); a study of this sort has not yet been conducted for glutamate. It is likely that this relationship between slope and uptake is different for glutamate, the levels of which are not only regulated by re-uptake via EAATs, but also by export via system X_c_^–^ (for further discussion, see Trantham-Davidson et al., 2012). Therefore, to directly examine the possibility that chronic ethanol may alter glutamate transporter function, we used an *ex vivo* preparation to measure the transport capacity for glutamate in ventral striatal tissue harvested from ethanol-dependent and non-dependent mice. Glutamate transporter function was assessed**** under Na^+^-dependent and -independent conditions to determine whether the excitatory amino acid transporter (EAATs) systems and system X_c_^–^, respectively, were influenced by ethanol dependence. The membrane bound EAATs are Na^+^-dependent and their role is to remove glutamate from the extracellular space, keeping extracellular glutamate low (Danbolt, 2001). On the other hand, system X_c_^–^ is Na^+^-independent and exchanges extracellular cysteine for intracellular glutamate, and contributes significantly to the extrasynaptic pool of glutamate (Baker et al., 2002). It is clear from our results that Na^+^-dependent transport constituted a much larger fraction of glutamate transport than did Na^+^-independent transport, a finding consistent with reports in rats (Melendez et al., 2005). Further, the specificity of the assay system was confirmed using the inhibitors TBOA and CPG to pharmacologically probe Na^+^-dependent and -independent transport function, respectively. Overall, our results indicate that function of these two important glutamate transport systems are not altered in our CIE model, at least in the NAc. This finding is in contrast to previous work (Melendez et al., 2005), showing that increased extracellular glutamate was associated with reduced transporter function. The reason for the discrepancy in results between the earlier study and the present one is unclear, but in addition to the use of different species, the differences could also be related to the amount of ethanol exposure and timing of the experimental measurements. Melendez et al. (2005) administered daily injections of 1 g/kg ethanol for 1 week whereas the CIE exposure procedure produced blood ethanol concentrations of ∼200 mg/kg that were sustained for 16 h, four times per week continuing for 3–4 weeks in the present study. Additionally, whereas the previous study conducted measurements 24 h after discontinuing ethanol exposure (Melendez et al., 2005), the current study used a 72 h time point because it coincides with a time when mice are allowed to resume voluntary drinking and signs of physical withdrawal from ethanol have abated (Becker and Hale, 1993). In fact, a very recent study found differences in the timing of specific physical withdrawal symptoms between two strains of rats exposed to high doses of ethanol (Abulseoud et al., 2014), suggesting that different ethanol exposure models may result in temporally distinct patterns of functional and/or expression changes in relevant proteins. Therefore, the amount of ethanol exposure as well as the timing of measurements relative to the initiation of withdrawal may be important variables to examine in future studies.

Taken together, results from the quantitative microdialysis and transport assay experiments indicate that glutamate transport function in the NAc is not altered by CIE exposure in this model of ethanol dependence. In the present study these measurements were determined at 3 days (72 h) following final CIE exposure. While we have previously reported that repeated cycles of CIE exposure produced elevated extracellular glutamate levels in NAc at least 6–7 days following the chronic ethanol treatment, it is not known whether alterations in glutamate transporter function contribute to this protracted effect. Future studies will need to determine the durability of this hyperglutamatergic state in NAc following CIE exposure. In addition, there is a possibility that expression of one or more of the transporter systems is altered. Despite large increases in extracellular glutamate following ethanol exposure, other reports indicate that EAAT2 (Melendez et al., 2005; Ding et al., 2013) and xCT (e.g., System X_c_^–^; Ding et al., 2013; Alhaddad et al., 2014a) expression in the NAc of rats was not significantly affected by ethanol exposure, although significant reductions in EAAT1 were reported (Ding et al., 2013). Other reports have found that EAAT2 expression in the NAc is significantly reduced in continuously drinking P rats (Sari and Sreemantula, 2012; Sari et al., 2013; Alhaddad et al., 2014a,b). Together, these findings underscore the complexity of the systems regulating glutamate homeostasis and suggest that alterations in one or more these proteins can be crucial to the outcome.

The mechanisms underlying elevated basal accumbal glutamate activity following CIE exposure are unknown. While action potential-dependent glutamate release does not ordinarily contribute to basal glutamate levels as measured by microdialysis (Baker et al., 2002), one possibility is that vesicular glutamate release may be increased after CIE exposure and this contributes to higher basal glutamate levels. In this situation, the increased extracellular glutamate in the would likely come from excitatory projections arising in the prefrontal cortex, amgydala, or hippocampus (e.g., Britt et al., 2012) that become hyperexcitable as a result of ethanol dependence. Another possibility is that expression or function of group II metabotropic receptors is altered. These receptors, mGluR2 and mGluR3, are found in the NAc (Ohishi et al., 1998; Tamaru et al., 2001). In addition, group II receptors play a crucial role in regulating extracellular glutamate concentrations because activating mGluR2/3 receptors reduces pre-synaptic glutamate release (Lovinger and McCool, 1995; Cartmell and Schoepp, 2000) and also reduces release of glutamate via system X_c_^–^ (Winder and Conn, 1996; Baker et al., 2002). Further, other reports have specifically implicated mGluR2 receptors in ethanol drinking by non-dependent (Zhou et al., 2013) and ethanol-dependent (Meinhardt et al., 2013) rats, making these receptors an important target for future work.

In summary, these data indicate that repeated cycles of CIE exposure disrupt glutamate homeostasis in the NAc, an important brain region implicated in motivated behaviors such as ethanol drinking. Specifically, in a model of ethanol dependence that produces escalated drinking, significant (twofold) elevation in basal extracellular glutamate concentrations in NAc was observed. Using an *ex vivo* assay to evaluate glutamate transporter function, CIE exposure did not alter Na^+^-dependent and -independent transporter functions. That is, CIE-induced elevation of basal glutamate levels in the NAc does not appear related to alterations in transport mechanisms responsible for bringing glutamate back into cellular compartments (i.e., Na^+^-dependent processes; EAATs) or mechanisms that release glutamate into the extracellular space (i.e., Na^+^-independent processes; system X_c_^–^). Given the importance of group II receptors in regulating extracellular glutamate concentrations, including pre-synaptic release, ongoing work is investigating the role of group II receptors on regulating extracellular glutamate as well as ethanol drinking in the CIE model.

### Conflict of Interest Statement

The Guest Associate Editor Justin Gass declares that, despite being affiliated to the same institution as authors William C. Griffin, Vorani S. Ramachandra, and Howard C. Becker, the review process was handled objectively and no conflict of interest exists. The authors declare that the research was conducted in the absence of any commercial or financial relationships that could be construed as a potential conflict of interest.
